# Plasma Proteome Profiling of Centenarian Across Switzerland Reveals Key Youth‐Associated Proteins

**DOI:** 10.1111/acel.70409

**Published:** 2026-02-08

**Authors:** Flavien Delhaes, Justine Falciola, Adar Hoffman, Stéphanie Carnesecchi, Stefano Cavalli, Armin von Gunten, Daniela S. Jopp, François R. Herrmann, Karl‐Heinz Krause

**Affiliations:** ^1^ Division and Department of Geriatrics and Rehabilitation University Hospitals of Geneva and University of Geneva Geneva Switzerland; ^2^ Institute of Psychology University of Lausanne Lausanne Switzerland; ^3^ Swiss Centre of Expertise in Life Course Research (LIVES) University of Lausanne Lausanne Switzerland; ^4^ Department of Education Medical University of Geneva Geneva Switzerland; ^5^ Centre of Competence on Ageing University of Applied Sciences and Arts of Southern Switzerland (SUPSI) Manno Switzerland; ^6^ Old‐Age Psychiatry Service, Department of Psychiatry University Hospital Lausanne Prilly Switzerland; ^7^ Foundation for Medical Research Geneva Switzerland

**Keywords:** aging, centenarian, healthspan, inflammation, metabolism, plasma, proteomics

## Abstract

Centenarians exhibit remarkable longevity and compression of morbidity making them an ideal population for uncovering proteins associated with successful aging. Using proteomics, we characterized the immune and cardiometabolic profiles of centenarians' plasma from the SWISS100 cohort. We identified 583 differentially expressed proteins (DEPs) by centenarians when compared with hospitalized geriatric patients (age 80–90 years) and younger healthy participants (age 30–60 years). We replicated the association of 23 proteins with a standard set of aging proteins (APs) developed by the Targeting Aging with Metformin (TAME) consortium. By comparing the centenarian signature to an independent centenarian proteomics study, we identified 135 DEPs in both studies with identical aging directions, establishing a robust set of APs in centenarians. Applying fractional polynomial regressions, we uncovered proteins with linear and non‐linear profiles associated with age and identified a subgroup of 37 proteins with a younger signature in centenarians. Protein–protein interaction and pathway enrichment analyses of 37 proteins point to programmed cell death, metabolic enzyme pathways, regulation of extracellular matrix stability, immune and inflammatory responses, and neurotrophic signaling pathways. This novel approach to aging research has uncovered new proteins and pathways, which may present promising targets to understand processes associated with longevity and healthy aging.

## Graphical abstract

We characterized the plasma proteomic profiles of centenarians from the SWISS100 cohort, identifying 583 differentially expressed proteins compared to younger and geriatric groups. Cross‐validation with the independent datasets NECS and the TAME consortium confirmed a robust aging signature, while fractional polynomial regressions revealed a specific subgroup of 37 proteins maintaining a “younger” profile in centenarians. These proteins implicate key pathways in longevity, including immune response, metabolic regulation, and extracellular matrix stability, offering potential targets for healthy aging.

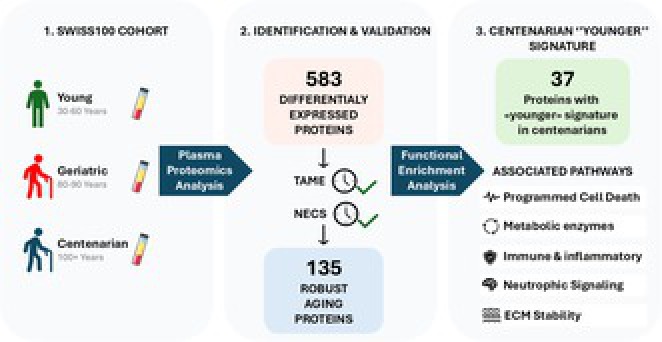

## 1 Introduction

### 1.1 Healthy Aging and Centenarians

The global number of “old(est)‐old” (people over age 85) including centenarians has been increasing substantially in the last half century and will continue to rise due to improved lifestyle and medical advances. By 2050, the European 85 years old and over population will double and the number of centenarians around the globe will reach nearly four million (Hernigou et al. 2024). Experts assume that in developed countries, half of the children born after the year 2000 will live to age 100 (Christensen et al. 2009). This increasing number of very old individuals represents a global demographic challenge, as aging is the largest risk factor for chronic disorders and increased vulnerability (Hernigou et al. 2024). The aging process varies significantly among individuals, with some facing severe disabilities and early mortality, while others maintain good health even when reaching advanced age. This discrepancy in the functioning and healthspan (e.g., period free from disease) observed in the aging population is mainly due to the complex interplay between intrinsic factors (e.g., longevity genes, telomere shortening, mutation accumulation, DNA repair capacity) and environmental factors (e.g., lifestyle, different access to proper nutrition, health care services, socioeconomic status) (Di Ciaula and Portincasa 2020). In response to the global aging population, the World Health Organization (WHO) has introduced the concept of healthy aging, defined as the process of maintaining the functional capacity necessary for older adults to preserve well‐being (Rudnicka et al. 2020).

To drive advancements in aging research, innovative strategies and discoveries are needed to promote health in older populations and prevent age‐dependent diseases and disabilities. Due to their extreme longevity and given that some of them may postpone diseases' apparition, centenarians have long been seen as prototypes of successful agers. Compared to the general population, they exhibit greater resilience and a markedly lower susceptibility to conditions such as cancers, diabetes, neurodegenerative disorders, or cardiovascular diseases than the normal population (Zhang et al. 2024). These unique characteristics have sparked growing research interest in uncovering the biological mechanisms and protective factors that contribute to healthy aging and longevity (Sebastiani et al. 2021; Takata et al. 1987). Previous studies have identified protective biological factors linked to longevity. In Okinawan centenarians, specific HLA alleles, particularly an increased HLA‐DR1 and lower HLA‐DRw9 frequency, appear to promote a less inflammatory immune profile (Takata et al. 1987). Mutations in the IGF‐1 receptor have been associated with exceptional longevity in Ashkenazi Jewish centenarians (Suh et al. 2008). Building on these genetic findings and the concept of age‐related inflammatory dysregulation (“inflammaging”) characterized in Italian centenarians (Franceschi et al. 2000), proteomic approaches now allow identification of downstream molecular signatures of healthy aging.

### 1.2 Proteomics & Blood

In recent years, proteomics has emerged as a powerful tool in identifying proteins involved in aging processes and those promoting longevity. The combination of proteomics analyses including mass spectrometry‐based, aptamers‐based (SomaScan), and Proximity Extension Assay (Olink) with large sample sizes (e.g., UK biobank) allows unprecedented levels of characterization of blood proteome for monitoring health in humans (Oh et al. 2025). Proteomics has been used to develop multiple proteomic aging clocks dependent on chronological age as well as identification of proteins independent of chronological age that may predict accelerated decline of health (Coenen et al. 2023). Oh et al. identified organ‐specific protein signatures associated with aging and diseases based on plasma proteomics (Oh et al. 2025). Other studies have developed proteomic signatures that correlate with health outcomes, revealing potential biomarkers for aging‐related conditions (Tanaka et al. 2020). Furthermore, the possibility to study thousands of proteins from the same samples not only allows for biomarkers identification, but also provides insights into the biological processes and pathways associated with aging and health maintenance (Sebastiani et al. 2021). These biomarkers may help monitor organ health and assess the efficacy of interventions aimed at extending the healthspan (Oh et al. 2025).

While proteomics offers promising avenues to identify healthspan‐related proteins, the complexity of aging and individual variability highlights the need for more studies on exceptional aging examples, such as centenarians, to elucidate the mechanisms of healthy aging. Santos‐Lozano et al. identified distinct proteins and pathways in healthy centenarians associated with enhanced immune function and lower inflammation. Their findings suggest that proteomics can reveal biological mechanisms underlying healthspan and successful aging, offering insights for future health interventions (Santos‐Lozano et al. 2020). The New England Centenarian Study (NECS) found that centenarian signatures were significantly enriched for senescence‐associated secretory factors (Sebastiani et al. 2021). Additionally, it identified two distinct serum protein signatures specific to extreme old age, highlighting unique proteomic features of longevity (Sebastiani et al. 2021). Still, proteomics data directly related to centenarians' blood is extremely scarce, and little is known about which proteins contribute to the protective effects during aging as well as the specific molecular mechanisms associated with successful aging.

In this study, we aimed to characterize the blood profile of centenarians by comparing their immune and cardiometabolic proteomic signatures to those of younger healthy participants (30–60 years) and hospitalized geriatric patients (80–90 years) from the SWISS100 study, using proximity extension assay. Specifically, we investigated whether centenarians possess key proteins that retain a level similar to younger participants (Frankowska et al. 2023) that may reveal key biological pathways related to a slower aging process and contribute to extended healthspan, enhanced resilience, or preserved functional capacity in older adults.

## 2 Results

### 2.1 Study Cohort Characteristics

SWISS100 participants were enrolled between November 2022 and December 2023, coming from three different linguistic parts of Switzerland and their samples were processed in three different sites (Geneva, Zurich, and Ticino) using the same protocol for blood collection, transport, processing and plasma storage. A flowchart (Figure S1) describes the steps applied to analyze proteomics data to:

- Discover plasma proteins whose expression differs between healthy and young participants (Healthy), hospitalized geriatric patients (Geriatric), and centenarians (Centenarian) and investigate their relation to aging protein biomarkers.
- Characterize age‐related linear versus non‐linear changes in protein levels.
- Identify key proteins in centenarians that are maintained at youthful levels and that may serve as indicators of healthy aging processes.

We plotted the global PCA results, coloring each data point by site, sex, or group for each participant (Figure S2, top). A distinct cluster was observed for the Zurich site, differing from Geneva and Ticino sites. Consequently, Zurich participants were excluded from the analysis (raw data including the three sites are available as a supplementary, see Table S6). Table 1 summarizes the demographic characteristics of the 40 healthy participants (mean age at blood draw 41 years), 55 hospitalized geriatric patients (mean age at blood draw 86 years), and 39 centenarians (mean age at blood draw 101 years) selected for this study. A detailed summary of the participants' diseases is provided in Table S1.

**TABLE 1:** Summary of participants' characteristics.

Characteristic	*N*	Centenarian, *N* = 39	Geriatric, *N* = 55	Healthy, *N* = 40	*p*
Site	134				< 0.001
Geneva		19 (49%)	55 (100%)	20 (50%)	
Ticino		20 (51%)	0 (0%)	20 (50%)	
Ethnicity	134				0.2
Asian		0 (0%)	1 (1.8%)	0 (0%)	
European Caucasian		39 (100%)	54 (98%)	38 (95%)	
Latino American		0 (0%)	0 (0%)	2 (5.0%)	
Gender	134				0.004
Female		33 (85%)	33 (60%)	20 (50%)	
Male		6 (15%)	22 (40%)	20 (50%)	
Age (years)	134	101 (101, 102)	86 (83, 88)	41 (34, 50)	< 0.001
Body mass index	120	24.2 (19.5, 26.8)	24.2 (21.6, 26.6)	22.9 (21.0, 25.0)	0.3
Unknown		12	2	0	
Systolic blood pressure (mmHg)	131	130 (107, 148)	135 (125, 147)	113 (106, 127)	< 0.001
Unknown		3	0	0	
Diastolic blood pressure (mmHg)	130	78 (69, 82)	69 (62, 75)	76 (72, 82)	< 0.001
Unknown		3	1	0	
Heart rate (bpm)	129	70 (64, 82)	79 (69, 88)	69 (62, 78)	0.003
Unknown		5	0	0	
General health	133				
1. Excellent		3 (7.7%)	1 (1.9%)	13 (33%)	
2. Very good		6 (15%)	17 (31%)	21 (53%)	
3. Good		20 (51%)	33 (61%)	6 (15%)	
4. Fair		7 (18%)	3 (5.6%)	0 (0%)	
5. Poor		3 (7.7%)	0 (0%)	0 (0%)	
Unknown		0	1	0	
Cigarette smoking	130				0.024
0. No, I have never smoked		30 (83%)	31 (57%)	22 (55%)	
1. Yes, but I have quit smoking		6 (17%)	21 (39%)	14 (35%)	
2. Yes, I currently smoke		0 (0%)	2 (3.7%)	4 (10%)	
Unknown		3	1	0	
Alcohol consumption	129				
0. No, never		12 (34%)	18 (33%)	1 (2.5%)	
1. No, not any more		2 (5.7%)	2 (3.7%)	0 (0%)	
2. Yes, a few times a month or less		10 (29%)	16 (30%)	23 (58%)	
3. Yes, a few times a week		5 (14%)	4 (7.4%)	15 (38%)	
4. Yes, most days		6 (17%)	14 (26%)	1 (2.5%)	
Unknown		4	1	0	
Physical activity	131				
1. Less than monthly		17 (46%)	15 (28%)	3 (7.5%)	
2. Monthly		0 (0%)	3 (5.6%)	1 (2.5%)	
3. Few times a month		1 (2.7%)	1 (1.9%)	5 (13%)	
4. Weekly		4 (11%)	5 (9.3%)	6 (15%)	
5. Few times a week		5 (14%)	9 (17%)	18 (45%)	
6. Daily		10 (27%)	21 (39%)	7 (18%)	
Unknown		2	1	0	
With pain	94	18 (46%)	19 (35%)	0 (NA%)	0.3
Unknown		0	0	40	
Pain frequency	87				0.6
1. Never		11 (33%)	14 (26%)	0 (NA%)	
2. Seldom		7 (21%)	14 (26%)	0 (NA%)	
3. Sometimes		9 (27%)	9 (17%)	0 (NA%)	
4. Frequently		3 (9.1%)	9 (17%)	0 (NA%)	
5. Always		3 (9.1%)	8 (15%)	0 (NA%)	
Unknown		6	1	40	

*Note:* Numbers represent counts for three groups (Centenarian, Geriatric, and Healthy). Numbers are median and interquartile range in parenthesis; median (Q1, Q3) or total numbers and percentage in parenthesis; *n* (%). Pearson's chi‐squared test; Fisher's exact test; Kruskal–Wallis.

### 2.2 Proteins Differentially Expressed in Centenarians

The protein profiles of the plasma samples were generated using a proximity extension assay that profiles 723 proteins, including proteins from an inflammation panel (357 proteins) and cardiometabolic panel (366 proteins). As three of them are shared by both panels (CXCL8, IL6, TNF), we examined a total of 720 unique proteins. Based on quality control analyses described in the methods section and Figure S1, we removed three outlier samples and analyzed the data of the remaining samples.

**FIGURE 1:**
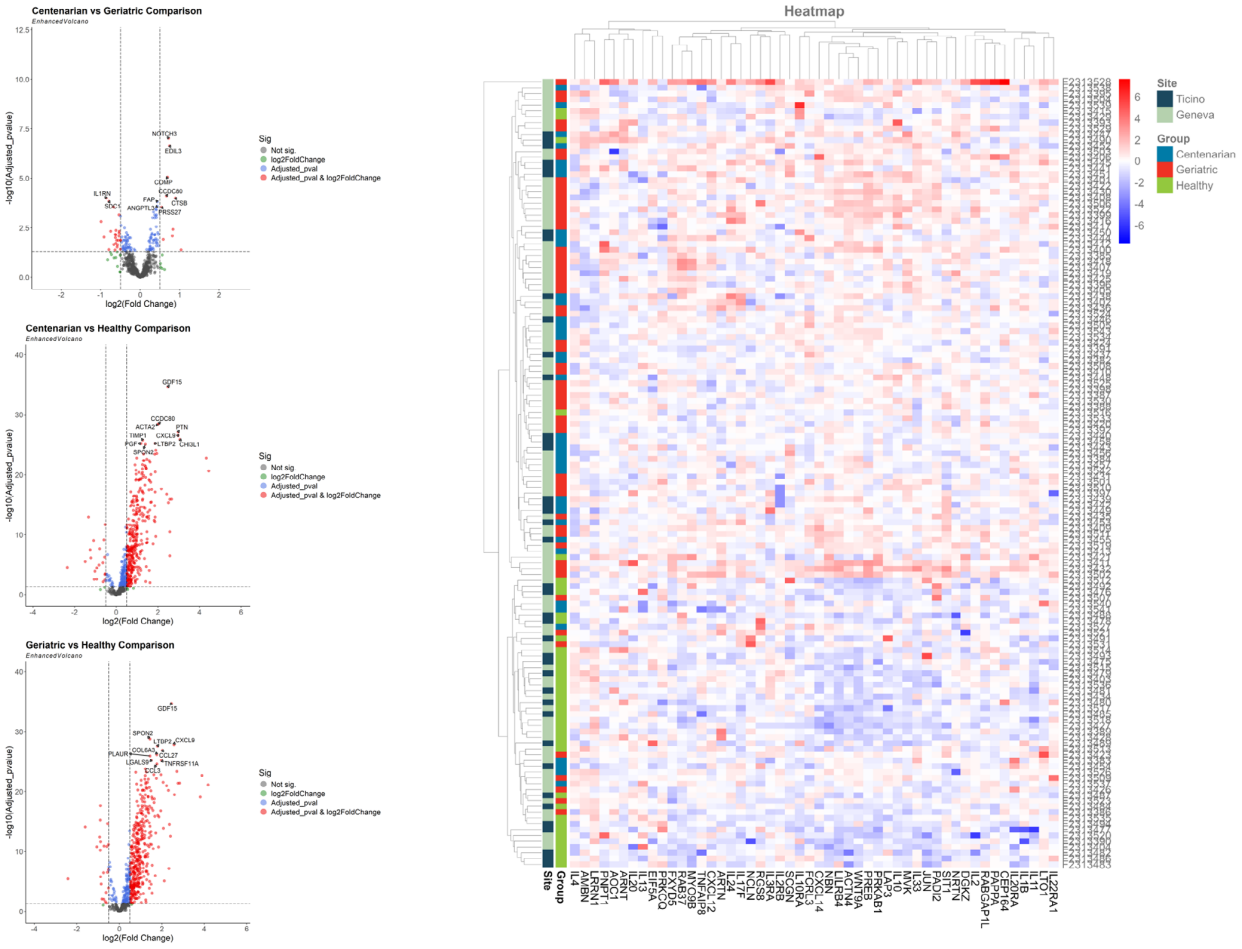
Volcano plots and heat map of differentially expressed proteins (DEPs) in centenarian, geriatric and healthy participants. Volcano plots (left), red dots represent proteins with a significant fold change (FC) ≥ 1.5 and adjusted‐*p* value < 0.05; green dots, proteins with FC ≥ 1.5 but not significant; blue dots, proteins with a FC ≤ 1.5 and adjusted‐*p* value < 0.05; and black dots, no meaningful changes in proteins. Name of 10 proteins with lowest adjusted‐*p* value are indicated in each plot. These results indicate that there is a significant difference in 583 protein levels expressed between centenarians, geriatric, and healthy. Heat map (right), the upregulated and downregulated DEPs are grouped by cluster analysis. The rows represent participants (centenarians, blue bar; geriatrics, red bar; healthy controls, green bar) and sites (Ticino, dark green; Geneva, light green). The columns represent the top 50 unique proteins with the lowest adjusted *p*‐value. The values in each cell represent log‐transformed normalized protein expression, red representing upregulation and blue representing downregulation. The full list of proteins with log2 FC and adjusted *p*‐value for all comparisons (Healthy2Cent, Cent2Geriatric, Healthy2Geriatric) is available in Table S2a–c.

We plotted the global PCA results, coloring each data point by site, sex or group for each participant (Figure S2, bottom). We did not observe clustering of data points for site or sex. Interestingly, we observed a separate cluster for the healthy participants compared to the geriatric and centenarian groups (Figure S2, bottom). Comparing the three groups using an ANOVA analysis, we identified 583 proteins with expression that significantly differed in centenarians. See Table S2 for the detailed lists of plasma proteins differentially expressed for each comparison (2a Centenarian—Healthy; 2b Centenarian—Geriatric; 2c Geriatric—Healthy). The volcano plots and heat map were used to depict the differences between the three groups (Figure 1). The heatmap in Figure 1, displaying an expression pattern for the top 50 proteins with the lowest *p*‐values, shows a distinct protein expression in healthy individuals compared to the geriatric and centenarian groups.

### 2.3 Comparison With the Targeting Aging With Metformin (TAME)‐Derived Panel of Aging Biomarkers

The full list of differentially expressed proteins was compared to the list of aging biomarkers in blood determined by the TAME working group (Justice et al. 2018), see full list of APs in Table S2d. Of the seven aging biomarkers selected by TAME as gold standard, five proteins (CST3, GDF15, IL6, NPPB, TNF) were available and significantly differentially expressed when comparing healthy controls with centenarians or with geriatric patients; two proteins (CRP, IGF1) were unavailable from the Olink panels (Cardiometabolic I and Inflammation I).

On the expanded list of blood‐based biomarkers (74 total) selected by TAME, when comparing healthy controls with centenarians, 47 biomarkers were not available in both panels (63.5%). Of the available markers, 23 were significantly differentially expressed in centenarians (85.2%) and 4 were not significantly differentially expressed in centenarians (14.8%). When comparing healthy controls with geriatric patients, 47 biomarkers were not available in both panels (63.5%); 25 were significantly differentially expressed in the geriatric group (92.6%), and only 2 were not significantly differentially expressed in hospitalized geriatric patients (7.4%). Only 2 proteins (SERPINE1, SOD1) among the 25 proteins available in the SWISS100 dataset demonstrated different results in both comparisons (Healthy2Cent and Healthy2Geriatric). Thus, both the short and expanded list of blood‐based biomarkers proposed by TAME as APs are highly reproducible in the SWISS100 study based on proximity extension assay. Table S2d contains the complete list of DEPs in blood with age that overlap between both studies.

### 2.4 Comparison With Proteomics Data From NECS

We compared the list of significant SWISS100 proteins to data from the NECS study (Sebastiani et al. 2021), an independent serum‐based dataset analyzed using the SomaScan platform. NECS included 77 centenarians (mean age 105.7), 82 offspring of centenarians (mean age 71.2), and 65 age‐matched controls (mean age 70.6), with protein profiles generated for 4116 unique proteins. We used FDR adjusted *p*‐values for both datasets and overlapped all significant proteins from the two studies (SWISS100 and NECS, see Table S2e) based on the provided UniProt identifier (155 overlapping proteins). Next, we determined the aging direction, that is whether proteins were up‐ or down‐regulated with age, by comparing the oldest‐old groups (centenarians or geriatric patients) with younger control groups (healthy controls or centenarians' offspring) for all overlapping proteins across the two studies and identified 135 proteins exhibiting aging effect with consistent directions; 12 proteins were significantly down‐regulated and 123 were up‐regulated, establishing a robust set of APs in centenarians. Table S2f contains the complete list of proteins over‐ and underexpressed in blood which overlap between both studies.

We also compared the list of SWISS100 proteins to data from the cross‐platform comparison performed by Reed et al., where a subset of 50 samples coming from NECS was used. to validate the SomaScan results based on mass spectrometry measures (Reed et al. 2024). The Reed comparison included: 4118 and 398 unique proteins for SomaScan and mass spectrometry, respectively. Of the 80 proteins quantified by both studies (SWISS100 and NECS‐mass spectrometry, see Table S2i), we identified 17 overlapping proteins based on FDR adjusted *p*‐values. Of these 17 proteins, 13 proteins exhibited aging effects with consistent directions across platforms; 1 protein was significantly down‐regulated and 12 were up‐regulated, establishing a specific set of APs in centenarians based on mass spectrometry results comparison. Table S2j contains the complete list of proteins over and underexpressed in the blood which overlap between both studies.

### 2.5 Fractional Polynomial Regression Reveals Linear and Nonlinear Age‐Associated Changes in Protein Trajectories

**FIGURE 2:**
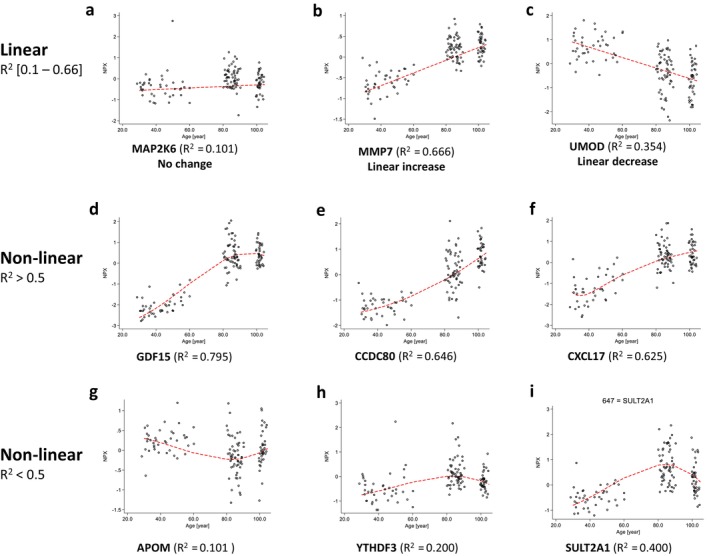
Fractional polynomials model for the association between age and protein level. Figure presents representative scatter plots with best‐fitting fractional polynomial regression association between age and protein level. Age (in years) was plotted against the logarithm of the protein level (in NPX); each dot indicates an individual participant. Two main fractional polynomial models were classified as linear association (up) with: no change; linear increase; linear decrease. Non‐linear association with the higher *R* ^2^ (middle) and lower *R* ^2^ (down), exact *R* ^2^ for the proteins was indicated for each graph. The full list of proteins with category association is available in Table S3.

As classic regression models assume a linear relationship between variables, we applied fractional polynomial (FP) modeling, a more flexible approach that effectively captures non‐linear associations, to examine the influence of aging on protein levels. We identified 149 proteins with no age‐related change, 30 proteins associated with linear decrease (*R* ^2^ = 0.02–0.35), 359 proteins associated with linear increase (*R* ^2^ = 0.02–0.67) and 182 proteins associated with non‐linear pattern (*R* ^2^ = 0.03–0.79) (Table 2). The top 10 proteins that most significantly correlated with age included Growth/differentiation factor 15 (GDF15), Insulin‐like growth factor‐binding protein 2 (IGFBP2), C‐X‐C motif chemokine 9 (CXCL9), Spondin‐2 (SPON2), C‐C motif chemokine 27 (CCL27), Latent‐transforming growth factor beta‐binding protein 2 (LTBP2), Actin, aortic smooth muscle (ACTA2), Matrilysin (MMP7), Collectin‐12 (COLEC12), Coiled‐coil domain‐containing protein 80 (CCDC80) (see Table S3 for the full list of proteins stratified and ranked from the most significantly to the less correlated with age). Interestingly, the top 10 proteins with the highest *R* ^2^‐values were predominantly associated with non‐linear patterns (9 on 10) including GDF15, one of the proteins most significantly up‐regulated during aging. Figure 2 illustrates the representative scatter plots with best‐fitting fractional polynomial regression association for each category. MAP2K6 was classified as linear with no change associated with age (Figure 2a), while MMP7 and UMOD demonstrated a linear increase pattern (Figure 2b) and linear decrease (Figure 2c) with age, respectively. GDF15 (Figure 2d) increased with age, but we observed a “break” in the curve when reaching 100 years old, indicating a decelerated aging process for this protein in centenarians. CCD80 (Figure 2e) expression increased exponentially with age, whereas CXCL17 (Figure 2f) exhibited stable expression until approximately 45 years of age, after which it shows a rapid increase in expression with age. Furthermore, some proteins with the lowest *R* ^2^ demonstrated other types of patterns like a “U‐shape” for APOM (Figure 2g), or an “inverted U‐shape” for YTHDF3, SULT2A1 (Figure 2h,i).

**TABLE 2:** Proteins summary categorized as linear increase, linear decrease, non linear based on fractional polynomials method.

	Total	No change	Linear decrease	Linear increase
Linear	538 (74.7%)	149 (20.6%)	30 (4.2%)	359 (49.9%)

	Total	*R* ^2^ > 0.5	*R* ^2^ < 0.5
Non‐linear	182 (25.3%)	39 (5.4%)	143 (19.9%)

*Note:* Table presents summary of protein number per category: Linear or Non‐linear based on fractional polynomials regression for the association between age and proteins. Linear association (up) was classified in three subcategories: proteins with no change, linear increase, linear decrease. Non linear association (down) was classified in 2 subcategories: proteins with *R* ^2^ > 0.5 and *R* ^2^ < 0.5. The full list of proteins in each category is available in Table S3.

### 2.6 Identification of Centenarian Youth‐Associated Proteins

Using fractional polynomial regression, we identified proteins demonstrating either accelerated or decelerated aging patterns. Since the centenarian signature may include proteins with levels similar to those of younger individuals, which may help centenarians preserve functional capacity and maintain a healthy phenotype, we focused on a subset of centenarian proteins that showed significant differential expression compared to the geriatric group but similar to the younger healthy group. We identified a 37‐protein signature in centenarians, characterized by expression levels similar to the younger and healthy group (Figure S3), see Table S2g for the distinct list of 37 centenarian youth‐associated proteins.

### 2.7 Protein–Protein Interaction and Pathway Enrichment of the Centenarian Youth‐ Associated Proteins List

To explore the specific biological functions associated with the 37‐centenarian youth‐associated proteins signature identified in centenarians, we constructed a protein–protein interaction (PPI) network using the STRING database and performed pathway enrichment analysis using the Reactome database, incorporating data from Kyoto Encyclopedia of Genes and Genomes (KEGG) and Gene Ontology (GO).

**FIGURE 3:**
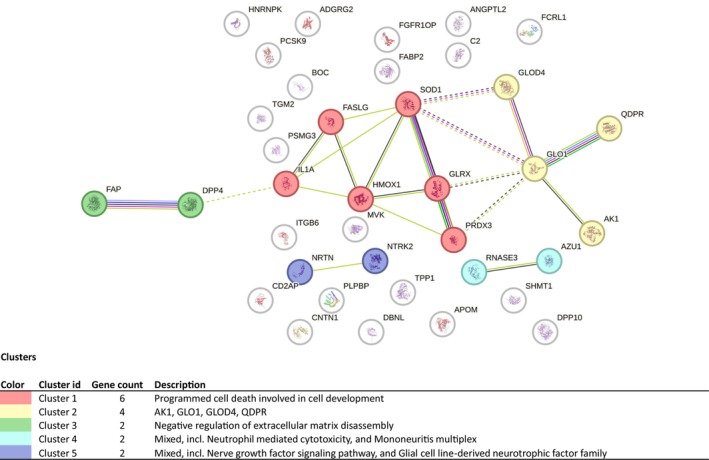
Protein–protein interaction (PPI) network representation for the centenarian youth proteins list. PPI network for the set of 37 proteins included in centenarian signature. It was constructed using STRING database with at least 2 node connections (link: http://string‐db.org, version 12.0). Required score: medium confidence (0, 4), FDR stringency (5%). Sub‐Network Corresponds to the Significant Cluster was determined by MCL clustering (inflation, 3).

Using the STRING database, we generated a PPI network (Figure 3). The constructed PPI network has 37 nodes and 22 connections, including 11 up‐regulated and 26 down‐regulated DEPs. We then applied MCL clustering and identified five clusters. These clusters were significantly enriched for “Programmed cell death involved in cell development”; “AK1, GLO1, GLOD4, QDPR”; “Negative regulation of extracellular matrix disassembly”; “Mixed, incl. Neutrophil mediated cytotoxicity, and Mononeuritis multiplex”; “Mixed, incl. Nerve growth factor signaling pathway, and Glial cell line‐derived neurotrophic factor family” terms. Top enriched GO terms from the STRING database included the “peptidase activity (serine‐type peptidase activity)”; “Antioxidant activity, and cell adhesion molecule binding” (Table S4a) for the entire network analysis.

In addition to PPI analysis, functional enrichment analysis of 37 DEPs was conducted using the Reactome pathway database (Table S5). The analysis identified 230 pathways, with 15 meeting the threshold of FDR < 0.01. seven proteins were removed as Reactome did not find them (FCRL1, ADGRG2, FAP, GLOD4, ANGPTL2, PLPBP, TGM2). These 15 pathways were categorized under six higher level pathways (HLPs): “Immune system”; “Cellular responses to stress”; “Signal transduction”; “Gene expression (Transcription)”; “Diseases”; “Metabolism”.

For the “*Immune System*” general pathway, the results showed that DEPs were significantly enriched in the following pathways: “Signaling by interleukins”; “Interleukin‐4 and Interleukin‐13 signaling”; “Cytokine Signaling in Immune system” with key proteins including IL1A, HMOX1, FASLG, and SOD1. For the “Cellular responses to stress” general pathway, enrichment was observed in the following pathways: “Regulation of HMOX1 expression and activity” and “NFE2L2 regulating anti‐oxidant/detoxification enzymes”, involving key proteins HMOX1 and SOD1. Additional pathways included “Detoxification of Reactive Oxygen Species”, with PRDX3 and SOD1 identified as key proteins. Other top results included “Cellular responses to stress”; “Cellular response to chemical stress” and “Cellular responses to stimuli”. Key hit proteins involved with these pathways include PRDX3, IL1A, HMOX1, TPP1, and SOD1. For the “Signal and transduction” general pathway, “Interconversion of Nucleotide Di‐ and Triphosphate” was enriched, with AK1 and GLRX as key proteins. For the “Gene expression (Transcription)” general pathway, the “FOXO‐Mediated Transcription of Cell Death Genes” was identified, including FASLG, which was also implicated in “Deregulated CDK5 triggers multiple neurodegenerative pathways in Alzheimer's disease models”; “Neurodegenerative Diseases”; “Defective Intrinsic Pathway for Apoptosis” that are part of “*Diseases*” general pathway. Finally, “Vitamin E” was found in the “*Metabolism*” general pathway with TPP1 highlighted as a key protein.

## 3 Discussion

### 3.1 Overview of the Results

The present study aimed at identifying proteins in centenarians' plasma that contribute to successful aging. Specifically, we analyzed circulating plasma proteins from healthy (~41 years), hospitalized geriatric (~86 years), and centenarian (~101 years) individuals based on proteomics. We identified 583 proteins with distinct expression profiles across the three groups, including 500 proteins differentiating centenarians from healthy participants and 163 proteins distinguishing them from hospitalized geriatric patients. The discovery of such a large number of significant proteins is likely due to the integration of extreme old age individuals compared to healthy and a hospitalized geriatric population that is unlikely to reach 100 years, as well as the type of proteins we targeted (inflammation and cardiometabolic panels). Alterations of the systemic inflammation and metabolic regulation will greatly disrupt key physiological functions including nutrient sensing, metabolic shifts, insulin sensitivity, cellular senescence, or inflammatory responses in most organs (Guo et al. 2022), ultimately accelerating the overall aging process.

Our proteomic findings provide molecular‐level insights that complement established genetic associations with longevity. Whereas genetic studies have identified protective HLA allele combinations that shape a less‐inflammatory immune environment (Takata et al. 1987) and IGF‐1 receptor mutations that confer metabolic advantages (Suh et al. 2008), our data show how these protective mechanisms may manifest at the protein level through the preserved, youth‐like expression of immune‐regulatory and metabolic molecules. These observations align with studies indicating that the centenarian proteome is qualitatively and quantitatively distinct from that of elderly individuals and reflects a slower intrinsic rate of aging (Frankowska et al. 2023; Sebastiani et al. 2021).

The initial list of DEPs identified in centenarian plasma from our study included well‐established aging biomarkers developed by the TAME consortium. Thus, the centenarian protein signature was enriched with APs and validated the use of proximity extension assay to identify differences between age groups. Furthermore, we identified nine proteins demonstrating age‐dependent characteristics (linear increase or decrease; Table 2) which were included in TAME extended list. This set includes CCL11, CST3, ICAM1, ICAM2, IFNG, IGFBP3, IL2, TGFB1, VCAM1 and represents proteins that reliably predict chronological age, reinforcing their potential use as hallmarks of aging. The presence of inflammatory markers (IL‐6, TNF, GDF15) among our validated TAME biomarkers strongly supports the inflammaging hypothesis (Franceschi et al. 2000), Importantly, these proteins showed a significant but attenuated increase in centenarians compared to hospitalized geriatric patients, suggesting preserved inflammatory regulation in exceptional longevity. By comparing our results with NECS, we validated a robust list of 135 DEPs with similar aging direction changes in centenarians compared to their respective control groups in both studies. These concordant results highlight the potential importance of these DEPs in understanding the differences in pathways between normal and exceptional aging.

Interestingly, we noted that a large number of proteins (49.9%) demonstrated a linear increase with age, while only a small fraction (4.2%) showed a linear decrease. This bias can be explained by the selection of the two Olink panels, as there is strong evidence in the literature that these proteins have an important role in aging and age‐related diseases (such as cardiovascular disease, cancer, and neurodegeneration). For the “Cardiometabolism panel”, since age is the biggest risk factor for these diseases, many of the selected proteins are naturally increasing with age, by design. For the “Inflammation panel”, this may relate to the “inflammaging” concept: as people age, the immune system tends to become dysregulated, leading to a persistent, low‐level inflammatory state, meaning many of the selected proteins are naturally increasing with age.

The 10 proteins most strongly correlated with age included established aging markers (GDF15, IGFBP2) and new candidates. IGFBP2 showed a notable age‐related exponential increase (*R* ^2^ = 0.67), consistent with its role in the IGF‐1 pathway, where receptor mutations are linked to longevity (Suh et al. 2008). CXCL9 (*R* ^2^ = 0.58) also emerged as a crucial inflammaging marker, supporting the inflammatory theory of aging (Franceschi et al. 2000).

The age‐related trajectories for nine of the top 10 proteins (highest *R* ^2^ values) were non‐linear, including SPON2 (spondin‐2, *R* ^2^ = 0.71) and CCL27 (*R* ^2^ = 0.69). This finding is consistent with the complex dynamics reported for Danish nonagenarian mortality predictors, where biomarker‐survival relationships also showed non‐linear characteristics (Nybo et al. 2003).

### 3.2 Novel Centenarian Youth‐Associated Proteins Signature

The identified proteins with non‐linear aging properties highlight the complex regulation of APs and emphasize the dynamic, non‐linear nature of the aging process. As a result, exploring nonlinear changes in biomarkers is likely to uncover previously unidentified protein signatures and provide new mechanistic insights. We hypothesized that certain proteins involved in maintaining physiological function in centenarians will persist at a youthful level, performing more effectively than in the average older adults (Frankowska et al. 2023). This is consistent with previous findings that a “younger” systemic environment and their circulating factors can slow down aging or even rejuvenate tissues (Liu et al. 2024). Of the 583 DEPs identified, 37 proteins in centenarians exhibit altered expression compared to the geriatric group, while resembling the expression patterns found in the younger group. These results show that centenarians carry a unique subset of 37 “youth‐associated proteins” that may point to important biological processes associated with healthy aging, as revealed by pathway enrichment and clustering analyses.

Interestingly, the Reactome analysis highlighted a significant enrichment of the “FOXO‐Mediated Transcription of Cell Death Genes” pathway. This enrichment reinforces the biological relevance of the final protein subset, as it aligns with the known importance of FOXO signaling in longevity (Morris et al. 2015) and the consistent role of FOXO3 as a pan‐ethnic longevity determinant (Willcox et al. 2008).

From the STRING analysis, we found a first cluster of proteins (FASLG, GLRX, HMOX1, IL1A, PRDX3, SOD1) that was enriched for the “programmed cell death” pathway in centenarians. Apoptosis is a well‐established contributor to age‐related changes (Guo et al. 2022), and the key proteins found in our study may contribute to maintaining healthy aging.

Similarly, Pinti et al. reported that Fas ligand (FASLG) mRNA levels were decreased in centenarians' lymphocytes, while Fas (FAS) mRNA levels were elevated when compared to young (~20 years) and middle‐aged participants (~60 years) (Pinti et al. 2004). By contrast, Borras et al. demonstrated an increase in both Fas and Fas ligand in the transcriptome of centenarians' peripheral blood mononuclear cells (PBMCs) compared with septuagenarians and young people (Borras et al. 2016). Furthermore, in progeroid Werner Syndrome patients, serum Fas ligand level was significantly higher than that in young healthy individuals (Goto 2008), which may indicate a potential role in an accelerated aging process. The conflicting results may be due to the nature of the samples, extraction type (PBMC, serum) or techniques used (RT‐PCR, transcriptome).

Glutaredoxin (GLRX), a marker of the redox balance, and heme oxygenase 1 (HMOX1), an enzyme with antioxidant and antiapoptotic effects, have never been described as differentially abundant in centenarians before. Still, they may play a role in the aging process, according to prior work. Glutaredoxin deletion was found to shorten the life span in *S. cerevisiae* (Liu et al. 2018), while the use of recombinant glutaredoxin increased lifespan and cellular stress resistance in *C. elegans* (Li et al. 2018). Ovarian expression of glutaredoxin 1 (Glrx1) significantly decreased with age in mice (Lim and Luderer 2011). HMOX1 transcript was twice as high in the PBMC of healthy aged participants (~75 years and over) compared to healthy young controls (~35 years) (Vo et al. 2011). Interestingly, HMOX1 was suggested to act as an anti‐aging molecule in human skin fibroblast (Lima et al. 2011) and can reduce age‐related inflammatory disease by regulating cellular senescence (O'Rourke et al. 2024).

Our findings suggest an association between interleukin 1A (IL1A) dysregulation and aging, which has been studied at the population genetics level. There was, for example, no significant association between IL1A genotype and longevity within a Finnish nonagenarian population (Wang et al. 2001) and an Italian centenarian population (Cavallone et al. 2003), but IL1Ra plasma levels were found to increase with age, indicating non‐genetic regulation. Interestingly, IL1A inhibition was suggested as a therapeutic approach to treat autoimmune and inflammatory diseases (Cavalli et al. 2021), and so contribute to extending the healthspan.

Peroxiredoxin 3 (PRDX3), an enzyme responsible for the detoxification of mitochondrial hydrogen peroxide, has never been described as differentially regulated in centenarians in prior work. Studies on non‐human models demonstrated, however, that PRDX3 may influence the fitness process. Inhibition of PRDX3 reduces steady‐state levels of ATP, motility, and brood size in *C. elegans* (Ranjan et al. 2013). Overexpression of PRDX3 in mice was shown to improve resistance to stress‐induced cell death and glucose homeostasis (Trujillo et al. 2023) and further reduce age‐related osteoarthritis, thus improving the healthspan (Loeser et al. 2022). Interestingly, other peroxiredoxins (PRDX1, PRX2, PRX4, PRX5) play a well‐defined role in regulating lifespan and survival across various aging model organisms (Nyström et al. 2012).

Superoxide dismutase 1 (SOD1), an antioxidant enzyme protecting the cell from reactive oxygen species toxicity, also seems to play a role in exceptional longevity, according to our findings. Studying influences on the aging process, previous studies demonstrated an association between SOD1*А/А genotype and longevity (Erdman et al. 2020), whereas another study did not (Gentschew et al. 2013). Interestingly, SOD activity was decreased in centenarian erythrocytes (Andersen et al. 1998). Additionally, Zhang et al. demonstrated that mice lacking SOD1 had significantly reduced lifespans and accelerated aging phenotypes (Zhang et al. 2017). Overexpression of SOD1 increased the life span in flies (Sun and Tower 1999) and *C. elegans* (Melov et al. 2000). Still, the involvement of SOD1 in human aging remains to be elucidated.

Importantly, Nuclear factor erythroid 2‐related factor 2 (NRF2), a master regulator of ~250 genes that target in particular HMOX1, SOD1, or PRDX present in our subset, was proposed as an approach to anti‐aging therapy by coordinating antiapoptotic and antioxidant responses (Zinovkin et al. 2022). This reinforces the importance of centenarian youth‐associated proteins identified in our study as potential targets for understanding the biological processes governing healthspan.

A second cluster of proteins (AK1, GLO1, GLOD4, QDPR) highlighted in our findings was enriched in the “energy metabolism” pathway. These proteins are important regulators of cellular homeostasis, glucose tolerance, or senescence and may impact healthspan. However, the roles and functions of the four proteins are poorly described in centenarians and deserve more attention in future studies.

More specifically, Adenylate kinase 1 (AK1) participates in the intracellular nucleotide metabolism homeostasis (AMP, ADP, ATP) and is one of the main regulators of AMPK. Many studies with lower organisms have revealed that increased AMPK signaling plays a significant role in aging: overexpression of AMPK gene or its chronic activation based on caloric restriction increases lifespan of worms (Apfeld et al. 2004) and *Drosophila* (Stenesen et al. 2013). Furthermore, numerous clinical trials are currently investigating the beneficial effects of AMPK activators, such as resveratrol, metformin, or exercise on human aging (Stancu 2015). Among the many products that can regulate AMPK, our study is the first to identify AK1 as potentially important for aging based on centenarian results.

Glyoxalase isoforms (GLO1, GLOD4), two enzymes involved in the removal of advanced glycation end products (AGEs), are present in higher concentrations in older adults and are associated with the development of chronic degenerative diseases related to aging (Luevano‐Contreras and Chapman‐Novakofski 2010). Overexpression of GLO1 in *C. elegans* decreases mitochondrial reactive‐oxygen species (ROS) production and extends lifespan, whereas GLO1 knockdown induces the exact opposite (Morcos et al. 2008). In mice, a GLO1 knockdown induces age‐related β‐cell dysfunction and glucose intolerance (Prevenzano et al. 2022), whereas overexpression improves neurovascular coupling and reduces cognitive impairment (Berends et al. 2024). Aragonès et al. suggest that targeting the modulation of the glyoxalase system could be a therapeutic approach to delay the onset of age‐related diseases (Aragonès et al. 2021). GLOD4 knockdown reduces lifespan in *C. elegans* with advanced neuronal damage and reduced motility with age (Chaudhuri et al. 2016).

Dihydropteridine reductase (QDPR) is an enzyme involved in the tetrahydrobiopterin BH4 regeneration. Dihydropteridine reductase deficiency causes hyperphenylalaninemia and neurological disorders, but its role in aging is still poorly understood. Use of high‐oleic peanut against aging and cognitive impairment reduced dihydropteridine reductase expression in the hippocampus of mice (Igarashi and Kurata 2020). Also, dihydropteridine reductase activity is changing over the course of life in humans and thus may have an important role in aging (Jeeps et al. 1986). Interestingly, BH4 is an essential cofactor required for nitric oxide (NO) production, and deficiency of BH4 may be involved in endothelial dysfunction related to aging (Higashi et al. 2006). Oxenkrug. proposed that interferon‐gamma inflammation participates in the downregulation of BH4 formation, representing a mechanism of aging (Oxenkrug 2011).

Our findings highlighted a third cluster, capturing the “Negative regulation of extracellular matrix disassembly” containing Prolyl endopeptidase (FAP) and dipeptidyl peptidase‐4 (DPP4). Both FAP and DPP4 are known to degrade proteins of the extracellular matrix. These peptidases are linked to inflammatory disorders and participate in pathological processes including type 2 diabetes mellitus (T2DM), fibrosis, arthritis, and cancer. Inhibition of FAP was shown to improve glucose and lipid metabolism in obese mice (Sánchez‐Garrido et al. 2016), whereas DPP4 inhibition improved glycaemic control in patients with T2DM (Deacon and Lebovitz 2016), making both of them interesting target for healthy aging, as confirmed by our results.

The fourth cluster (AZU1 and RNASE3) found was enriched for the “Mixed, incl. Neutrophil mediated cytotoxicity, and Mononeuritis multiplex” pathway. It is well known that chronic inflammation is increased in the aging population, as described by the “inflammaging” theory (Ferrucci and Fabbri 2018). Azurocidin 1 (AZU1), released by neutrophils, functions as a chemoattractant for monocytes and macrophages, potentially playing a key role in immune response activation and inflammation (Soehnlein and Lindbom 2009). The antimicrobial protein Ribonuclease A Family Member 3 (RNAS3) may have an important role in centenarian physiology. Sebastiani et al. already demonstrated that RNASE1 is part of the most differentially expressed proteins comparing centenarians with centenarians' offspring and controls (Sebastiani et al. 2021). Ribonuclease activities change with age in rats (Alberghina and Giuffrida Stella 1988). A study by Khalifah et al. demonstrated that glycation of ribonuclease A is a major contributor to the pathology of diabetes and possibly of aging‐related disorders (Khalifah et al. 1996). However, their precise molecular mechanisms in aging remain unknown and need more investigations.

The fifth cluster (NTRK2 and NRTN) highlighted in our findings was enriched for the “Mixed, incl. Nerve growth factor signalling pathway, and Glial cell line‐derived neurotrophic factor family” pathway.

Neurotrophic tyrosine receptor kinase (NTRK2) is part of the tyrosine kinase receptor family with broad biological roles. More specifically, NTRK2 was found to be associated with severe early‐onset obesity (Philippe et al. 2015) and age‐related cognitive decline (Tomás et al. 2023). Interestingly, inhibition of anaplastic lymphoma kinase (Alk), a protein that is part of the RTK family, has been shown to extend healthy lifespan in female *Drosophila* and preserve neuromuscular function with age (Woodling et al. 2020).

At the same time, neurturin (NRTN), a member of the glial‐derived neurotrophic factor family, demonstrated in prior studies some antidiabetic properties when infused in Zucker diabetic fatty (ZDF) rats (Trevaskis et al. 2017). A clinical trial using AAV‐neurturin gene (Cere120) improved the clinical status (improve motor function and reduce dyskinesia) in Parkinson disease patients (Marks et al. 2008). Our findings on the differential expression of NTRK2 and NRTN suggest that these proteins may exert a key influence on the progression of age‐related metabolic and neurological diseases.

Taken together, the 37 youth‐associated proteins converge on immune regulation and oxidative stress pathways, linking genetic predisposition to observed phenotypes in centenarians. The FOXO‐regulated cluster (FASLG, GLRX, HMOX1, IL1A, PRDX3, SOD1) mirrors longevity‐associated FOXO3 variants (Willcox et al. 2008). These enriched pathways support the inflammaging model (Franceschi et al. 2000) while suggesting active maintenance of anti‐inflammatory mechanisms beyond constitutive genetic protection.

### 3.3 Limitations

The present study provides new insights into proteins that may serve as biomarkers for longevity and healthy aging, as we applied strict exclusion criteria for healthy‐young participants and focused on centenarians. However, the study has limitations including a rather low number of participants and the unequal site and sex distribution in the age groups. We did not correct the analysis for sex, as no clustering for the sex variable was identified based on PCA results (Figure S2). Larger studies may be necessary to discriminate potential sex differences in protein expressions with protective aging effects. Another limitation is that we focused on a restricted number of proteins corresponding to two panels (i.e., inflammation and cardiometabolism) to conduct pathway enrichment analysis. This number was smaller than the plasma proteome, thus it excluded parts of biological pathways that could be evaluated based on Olink technology. Expanding the analysis by using the complete set of panels or other technologies could reveal new significant biological processes. Finally, while our findings establish a distinct proteomic signature across the groups, the interpretation is moderated by the substantial heterogeneity observed in the plasma profiles for Zurich participants. This site‐specific variation suggests that the underlying biological mechanisms are complex, potentially reflecting differences in population genetics or diverse environmental backgrounds unique to the Zurich cohort. Future work integrating genomic and lifestyle data would therefore be necessary to better understand the sources of this localized heterogeneity.

## 4 Conclusion

Proteomics offers a promising approach to identify proteins that have the potential to enhance the healthspan by revealing key biological processes involved in aging and health maintenance. Through the development of tools such as the proteomic aging clock, as well as gaining further insights on exercise‐related, caloric restriction, or anti‐aging therapies on proteomic patterns, researchers may be able to better understand the molecular mechanisms that promote a healthier and longer life. However, the complexity of aging and individual variability may pose challenges in translating these findings into universal biomarkers or therapies. Further research is needed to validate these proteins across diverse populations and conditions.

To the best of our knowledge, this study is the first to quantify 720 proteins associated with inflammation and cardiometabolism using a proximity extension assay in centenarians. Importantly, many of the identified centenarian youth‐associated proteins are involved in biological pathways that are consistent with current aging theory and extend beyond genetic predisposition. Previous studies identified genetic factors that promote longevity, including protective HLA alleles (Takata et al. 1987), IGF‐1 receptor mutations (Suh et al. 2008), and FOXO3 variants (Willcox et al. 2008), our findings demonstrate that centenarians also maintain youthful protein expression patterns in critical pathways. The 37 youth‐associated proteins, particularly those involved in immune regulation and oxidative‐stress response, suggest that successful aging reflects both inherited protective factors and active maintenance of molecular homeostasis. The non‐linear protein trajectories observed here and in other cohorts (Nybo et al. 2003) indicate that aging biomarkers may require age‐specific interpretation, particularly in extreme longevity. Integrating genomic and proteomic data across diverse centenarian populations will be crucial for distinguishing constitutive genetic advantages from acquired resilience, improving our understanding of aging and guiding future therapies.

## 5 Experimental Section/Methods

### 5.1 Study Population

SWISS100 participants were enrolled between November 2022 and December 2023, coming from different cantons with different languages (French: Geneva and Vaud; German: Basel, Bern and Zurich; Italian: Ticino) and processed in three different sites (Geneva, Zurich, and Ticino) using the same protocol for blood collection, transport, processing and plasma storage. All participants provided informed written consent for the study under research project 2021‐02509, which was approved by Swiss Ethics Committees at each site. The three cohorts included a total of 382 participants: inclusion criteria encompassed an age between 30 and 60 years for the healthy group (*n* = 139), 80–90 years for hospitalized patients part of the geriatric group (*n* = 116) and 100 years and older for the centenarian group (*n* = 127). For the healthy participants, exclusion criteria encompassed current or history of any significant medical disorder such as inflammatory, metabolic, auto‐immune, coagulation, unstable cardiac or respiratory diseases. All participants underwent a questionnaire to evaluate medical history (ICD‐10 coded), health status and demographic factors (age, sex, ethnicity, BMI, smoking status, alcohol consumption, physical activity, pain and pain frequency). Blood pressure was measured twice, and averaged values were used. Demographic data and medical history are presented in Table 1, Table S1, respectively. Participant underwent a one‐time blood collection.

### 5.2 Samples

Venous blood was collected for proteomic measurements with a tourniquet in BD P100 (Becton Dickinson, ref. 366448) tubes which contain K2EDTA additive and proprietary protein stabilizers. Tubes were transported at 4°C to minimize protein degradation. Blood was then centrifuged at 2000×*g* for 10 min at room temperature (RT). Plasma supernatants were aliquoted (500 μL final volume) in new tubes (Axygen, ref. MCT‐060‐C) on ice and stored at −80°C.

### 5.3 Proteomics

Plasma proteins were measured in 176 participants randomly selected from the original SWISS100 study. The plasma samples were measured at the Firalis facility in Huningue, France. All samples were randomized and plated by the Firalis laboratory team. The concentrations of 723 proteins (Table S2) were measured using the Olink Explore platform based on Proximity extension Assay (PEA) technology. The proteins were preselected from two panels: Olink Explore 384 Cardiometabolic I and 384 Inflammation I. NovaSeq 6000 Sequencing Systems was used to quantify the protein concentration that was converted into Normalized Protein eXpression (NPX) for inter‐sample comparability. NPX is Olink's relative protein quantification unit on a log_2_ scale. 3 of 723 total proteins analyzed are shared by both panels (CXCL8, IL6, TNF) giving us a total of 720 unique proteins. Quality control measures and normalization of protein concentration were performed at the Firalis facility. We excluded participants who had outliers (*n* = 3) or site bias (*n* = 40), resulting in an analytic sample of 134 participants.

### 5.4 Identification of DEPs

Analysis of variance (ANOVA) was used to identify DEPs between the three groups (Healthy, Geriatric, and Centenarian). Identification of DEPs using volcano plots and heatmap for group comparisons. Multi‐test correction was performed using the Benjamini‐& Hochberg method, and proteins were defined as significantly differentially expressed if they had a Benjamini‐& Hochberg False Discovery Rate (FDR) adjusted *p*‐value below 0.05. Statistical analyses were performed in “R (R version 4.0.0, http://www.r‐project.org)”. We used the ANOVA function from the Olink package which applied systematically the Benjamini‐Hochberg correction by multiplying the classical *p*‐values by an appropriate correcting factor.

### 5.5 Identification of Proteins Linear and Non‐Linear Changes During Aging

We performed fractional polynomial regressions to test for the effect of age on protein expression levels. Proteins were defined as significantly associated to aging at a Benjamini‐Hochberg (FDR) adjusted *p*‐value below 0.05. Statistical analyses were performed with Stata, version 18.5 software (StataCorp, College Station, TX, USA).

### 5.6 Identification of Centenarian Youth‐Associated Proteins Signature

Due to variability in the rate of aging in measured proteins across groups, we identified all proteins significantly differentially expressed with age between the centenarian and geriatric groups with FDR adjusted *p*‐value below 0.05, but similar to healthy group. Centenarians' proteins similar to healthy groups were selected based on pairwise comparisons (Tukey's test) to identify those where expression levels in the Centenarian group were not significantly different from the Healthy group (*p* > 0.05, FDR‐adjusted) but were significantly different from the Geriatric group (*p* < 0.05, FDR‐adjusted). Statistical analyses were performed in R (4.4.0).

### 5.7 Protein–Protein Interaction Networks

We obtained Protein–Protein interaction (PPI) networks using the STRING database with at least 2 node connections (http://string‐db.org, version 12.0). 37 centenarian youth‐associated proteins were mapped based on their UniProt ID. Network Type: full STRING network; a medium confidence (0, 4); FDR stringency (5%) criteria were used for the network creation.

### 5.8 Cluster Analysis of the Plasma Proteome and Functional Enrichment of Younger Centenarian Protein Signature

An unsupervised hierarchical clustering analysis was performed using the MCL function from the STRING website (inflation, 3). To identify the biological relevance of our protein subsets of interest, we mined the KEGG, GO, and Reactome databases using the STRING database.

### 5.9 Pathway Enrichment Analysis of Younger Centenarian Protein Signature

A total of 37 “younger centenarian proteins” were mapped based on their UniProt ID using the Reactome tool to identify the differential pathways and molecular processes related to the healthy centenarian's aging. This approach employs a hypergeometric model to evaluate whether the number of selected genes linked to a Reactome pathway exceeds the expected count. A significance threshold was defined at FDR adjusted *p*‐value < 0.01.

## Author Contributions

Flavien Delhaes, Stéphanie Carnesecchi, Stefano Cavalli, Armin von Gunten, Daniela Jopp, François Herrmann, and Karl‐Heinz Krause contributed to study design, analysis plan, interpretation of the results, and drafting of the manuscript. Flavien Delhaes, Justine Falciola, Adar Hoffman, François Herrmann conducted the analyses. All authors contributed to the final version of the manuscript.

## Funding

This work was supported by the Swiss National Science Foundation (SNSF) [grant numbers: CRSII5_186239/1].

## Conflicts of Interest

The authors declare no conflicts of interest.

## Supporting information


**Figure S1:** Schematic breakdown of the methodology used in SWISS100 study. Total number of proteins after selection are indicated in yellow circle for each step. Identified Aging proteins (APs) in SWISS100 were compared with TAME and NECS studies and overlapping proteins selected based on the provided UniProt identifier. We used fractional polynomial regression to determine the association between age and proteins level. We applied STRING and Reactome analyses on centenarian biomarker signatures for pathway and functional enrichment.

**Figure S2:** Two‐dimensional PCA clustering plot for SWISS100 participants. Figure presents two‐dimensional PCA clustering plot for site, sex and group, each dot indicates an individual participant from SWISS100 cohort. (Top) PCA for 3 sites (Geneva‐Ticino‐Zurich), (Bottom) PCA for 2 sites (Geneva‐Ticino).

**Figure S3:** Plasma protein levels for the 37 proteins included in the centenarian youth proteins list. The analysis was performed on ANOVA; proteins significantly different between centenarians and geriatric but similar to healthy individuals were selected. Violin plot showing the relative concentrations between the three groups (healthy, geriatric and centenarian). Bottom and top lines of the boxes depict 25th and 75th percentiles; each dot indicates an individual participant. Log2 fold change and significance levels between groups were calculated using the ANOVA *F*‐test. If *p* < 0.05, paired comparisons were conducted with the estimated marginal means. Multiple testing correction was performed using the Benjamini–Hochberg method and a 5% FDR used for the significance threshold. For the final list of centenarian youth proteins with log2 FC and adjusted *p*‐value for comparisons with Cent2Geriatric see Table S2g.

**Table S1:** Summary of participants' diseases.
Numbers represent disease counts for three groups (Centenarian, Geriatric and Healthy) in ICD‐10 chapter. Numbers are total numbers and percentage in parenthesis; *n* (%). Fisher's exact test.

**Table S2:** Results of differential expression analysis using ANOVA for 2 sites (Geneva, Ticino). 2a Centenarian—Healthy. log2FoldChange: Fold change (FC) comparing protein abundance in centenarian versus healthy controls. FC Centenarian—Healthy > 0 indicates a protein expression that increases in the Centenarian group, whereas FC Centenarian—Healthy < 0 indicates a protein expression that decreases in the Centenarian group. Adjusted *p*‐values were computed using the Benjamini‐Hochberg correction. 2b Centenarian—Geriatric. log2FoldChange: Fold change (FC) comparing protein abundance in centenarian versus geriatric. FC Centenarian—Geriatric > 0 indicates a protein expression that increases in the Centenarian group, whereas FC Centenarian—Geriatric < 0 indicates a protein expression that decreases in the Centenarian group. Adjusted *p*‐values were computed using the Benjamini‐Hochberg correction. 2c Geriatric—Healthy. log2FoldChange: Fold change (FC) comparing protein abundance in geriatric versus healthy controls. FC Geriatric—Healthy > 0 indicates a protein expression that increases in the Geriatric group, whereas FC Geriatric—Healthy < 0 indicates a protein expression that decreases in the Geriatric group. Adjusted *p*‐values were computed using the Benjamini‐Hochberg correction. 2d Comparison of biomarkers selected by the TAME consortium with centenarians‐healthy controls and geriatric‐healthy controls. FC, adjusted *p*‐value and threshold for cent2healthy and geriatric2healthy were reported from Table S2a,c. 2e Comparison of biomarkers selected from SWISS100 cohort with NECS cohort. FC, adjusted *p*‐value and threshold for cent2healthy (SWISS100) and geriatric2healthy (SWISS100) were reported from Table S2a,c and FC, adjusted *p*‐value and threshold for cont2cent (NECS) were reported from Sebastiani supplementary. 2f Robust list of proteins overexpressed and underexpressed in centenarian from SWISS100 study that overlap with in centenarian from NECS study. UniProt identifier was used to identify overlapping proteins between all proteins in both studies (SWISS100 and NECS). 155 proteins with significant differences (FDR < 0.05) between groups (Cent2Healthy; Geriatric2Healthy, Cont2Cent) in 2 different studies (SWISS100 and NECS) were overlapping, of which 135 APs showed similar age‐associated effects (up or down) across all studies. 2g Final list of 37 Centenarian youth proteins. Final list of 37 proteins with gene symbol, UniProt, protein direction in centenarian compared to geriatric, log2FoldChange and adjusted *p*‐value. 2h Youth proteins determined in SWISS100 study also available from Sebastiani paper. 2i Comparison of biomarkers selected from SWISS100 cohort with NECS‐Mass spectrometry. FC, adjusted *p*‐value and threshold for cent2healthy (SWISS100) and geriatric2healthy (SWISS100) were reported from Table S2a,c and FC, adjusted *p*‐value and threshold for cont2cent (NECS‐Mass spectrometry) were reported from Reed supplementary. 2j Final list of 13 proteins showing similar age‐associated effects across studies SWISS100 and NECS‐Mass spectrometry, with gene symbol, UniProt, protein direction in centenarian compared to geriatric.

**Table S3:** List of proteins categorized as linear increase, linear decrease, non‐linear based on fractional polynomials method. Table presents a list of proteins categorized in three categories (linear increase, linear decrease, non‐linear) based on fractional polynomials regressions for the association between age and proteins level. *R* ^2^ and fractional polynomials equations were indicated for each protein.

**Table S4:** String pathway enrichment analysis results for the centenarian youth proteins list. Detailed results for GO Function, GO Component, STRING clusters enrichment for the set of 37 proteins included in centenarian signature.

**Table S5:** Reactome pathway enrichment analysis results for the centenarian youth proteins list. Detailed results for pathway enrichment for the set of 37 proteins included in centenarian signature.

**Table S6:** Results of differential expression analysis using ANOVA for 3 sites (Geneva, Zurich, Ticino). 2a Centenarian—Healthy. log2FoldChange: Fold change (FC) comparing protein abundance in centenarian versus healthy controls. FC Centenarian—Healthy > 0 indicates a protein expression that increases in the Centenarian group, whereas FC Centenarian—Healthy < 0 indicates a protein expression that decreases in the Centenarian group. Adjusted *p*‐values were computed using the Benjamini‐Hochberg correction. 2b Centenarian—Geriatric. log2FoldChange: Fold change (FC) comparing protein abundance in centenarian versus geriatric. FC Centenarian—Geriatric > 0 indicates a protein expression that increases in the Centenarian group, whereas FC Centenarian—Geriatric < 0 indicates a protein expression that decreases in the Centenarian group. Adjusted *p*‐values were computed using the Benjamini‐Hochberg correction. 2c Geriatric—Healthy. log2FoldChange: Fold change (FC) comparing protein abundance in geriatric versus healthy controls. FC Geriatric—Healthy > 0 indicates a protein expression that increases in the Geriatric group, whereas FC Geriatric—Healthy < 0 indicates a protein expression that decreases in the Geriatric group. Adjusted *p*‐values were computed using the Benjamini‐Hochberg correction.

## Data Availability

NPX data is available in Supporting Information and Zenodo 10.5281/zenodo.18403090. The digitally shareable code and data necessary to reproduce the reported results are available on GitHub https://github.com/fdelhaes/SWISS100.
