# Supplementary figures and images for: Plasma Proteome Profiling of Centenarian Across Switzerland Reveals Key Youth‐Associated Proteins
Source: Aging Cell. 2026 Feb 8;25(2):e70409. doi: 10.1111/acel.70409 (PMC12883144; doi:10.1111/acel.70409)

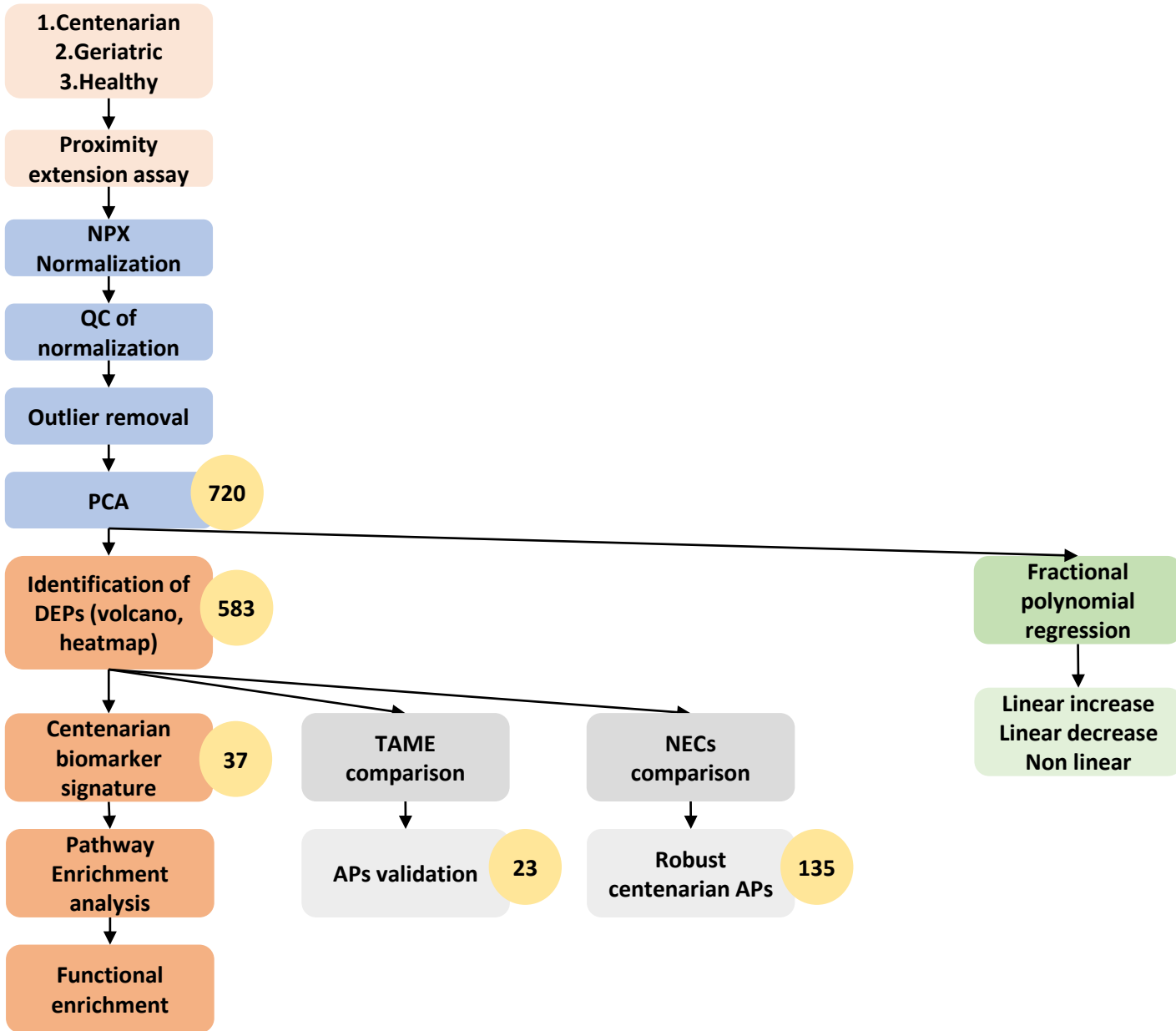

Supplement: Supplementary file 1 — Figure S1: Schematic breakdown of the methodology used in SWISS100 study. Total number of proteins after selection are indicated in yellow circle for each step. Identified Aging proteins (APs) in SWISS100 were compared with TAME and NECS studies and overlapping proteins selected based on the provided UniProt identifier. We used fractional polynomial regression to determine the association between age and proteins level. We applied STRING and Reactome analyses on centenarian biomarker signatures for pathway and functional enrichment. [file ACEL-25-e70409-s001.pdf]

### 3 sites Geneva-Ticino-Zurich

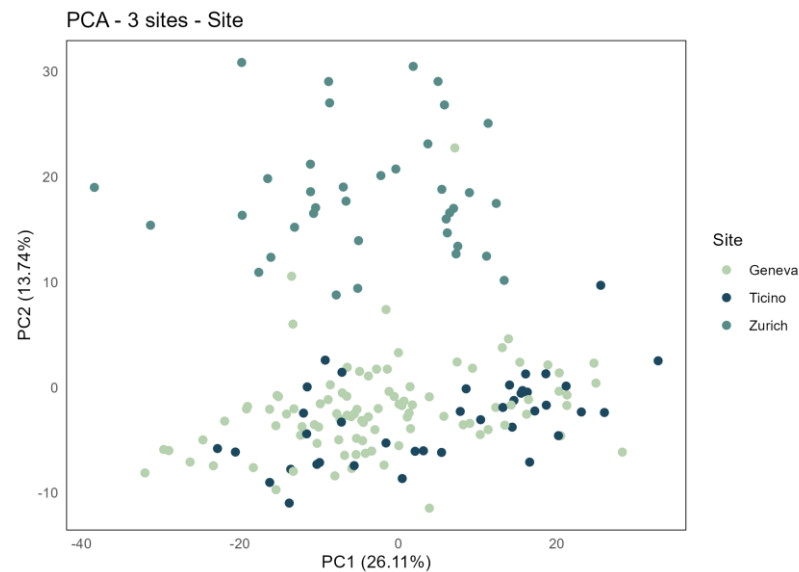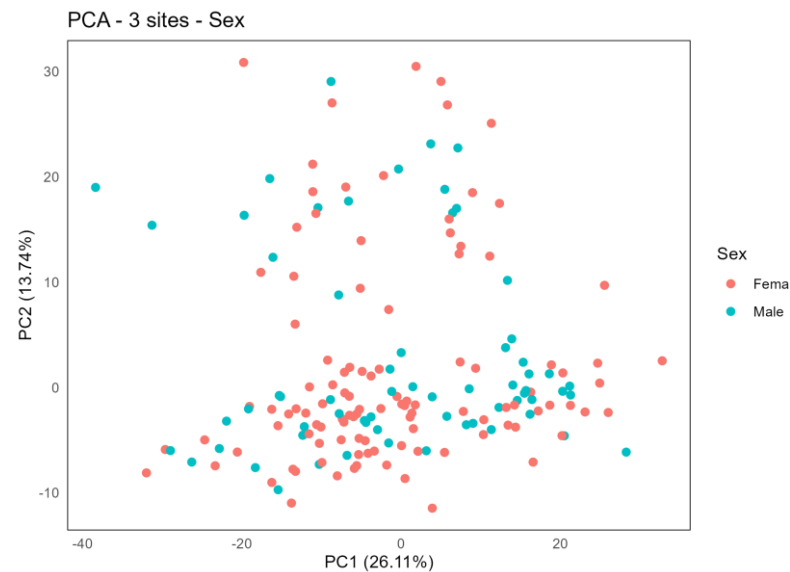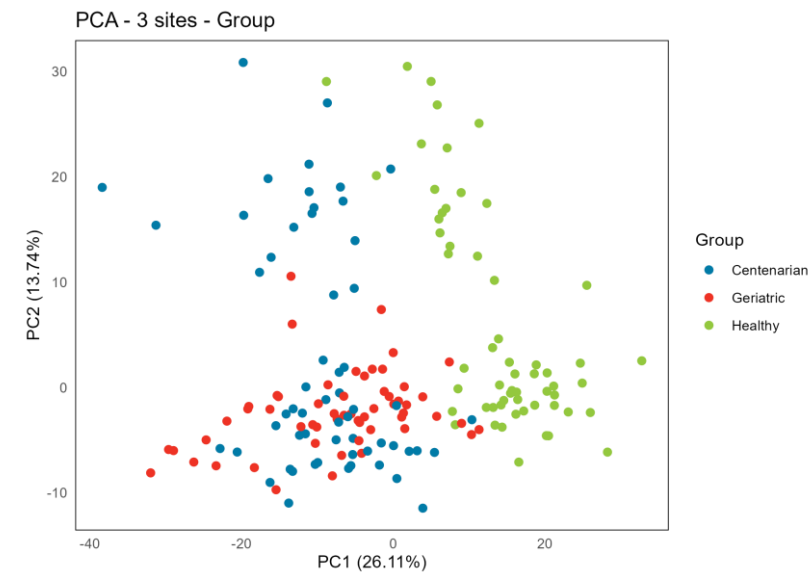

### 2 sites Geneva-Ticino

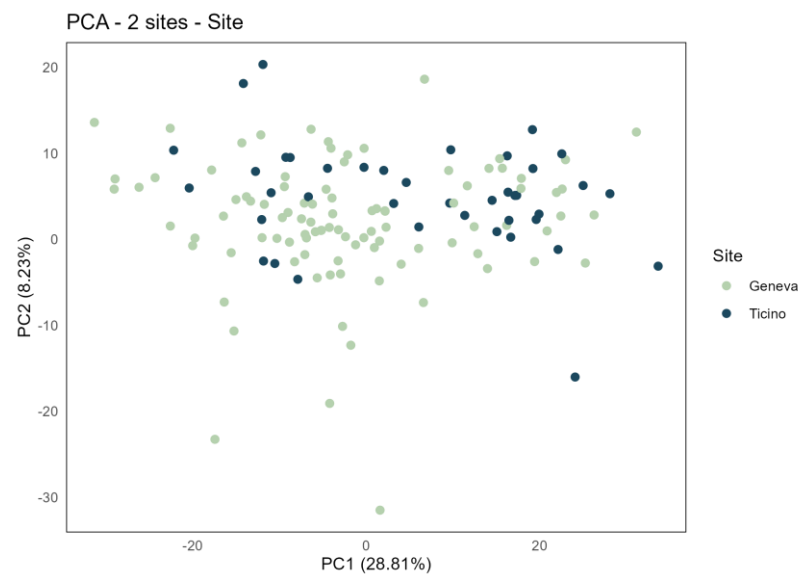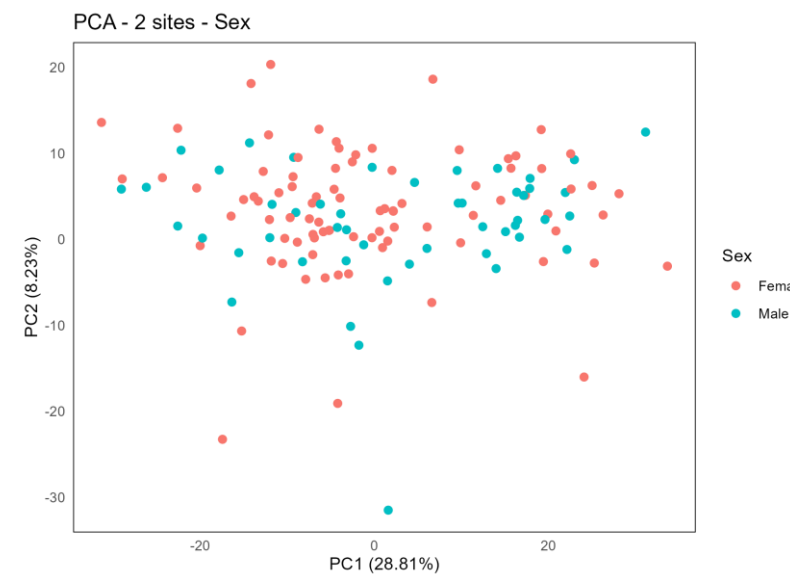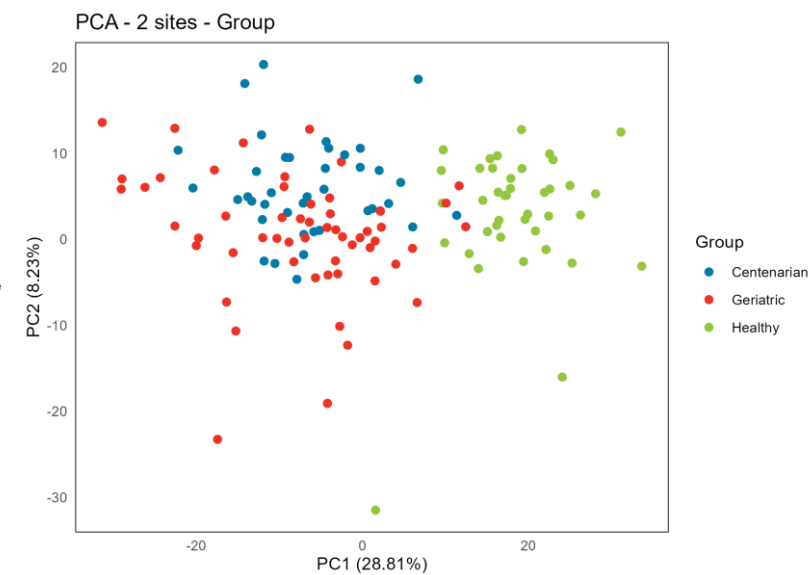

Supplement: Supplementary file 2 — Figure S2: Two‐dimensional PCA clustering plot for SWISS100 participants. Figure presents two‐dimensional PCA clustering plot for site, sex and group, each dot indicates an individual participant from SWISS100 cohort. (Top) PCA for 3 sites (Geneva‐Ticino‐Zurich), (Bottom) PCA for 2 sites (Geneva‐Ticino). [file ACEL-25-e70409-s009.pdf]

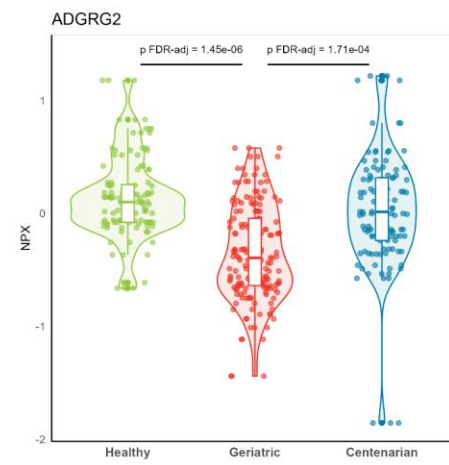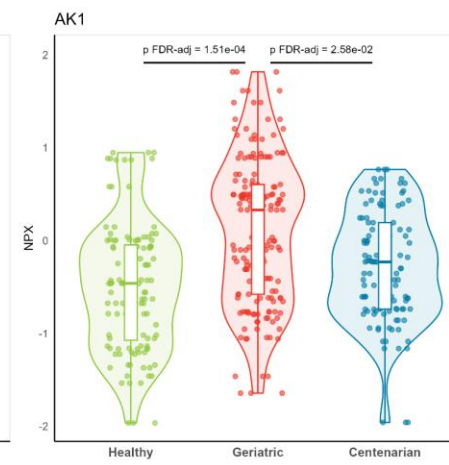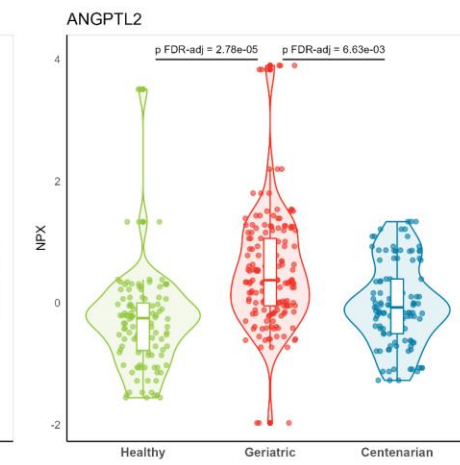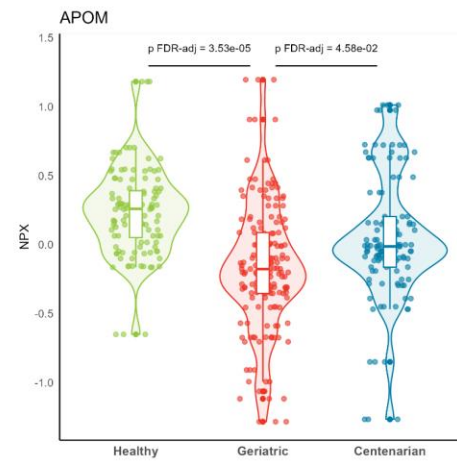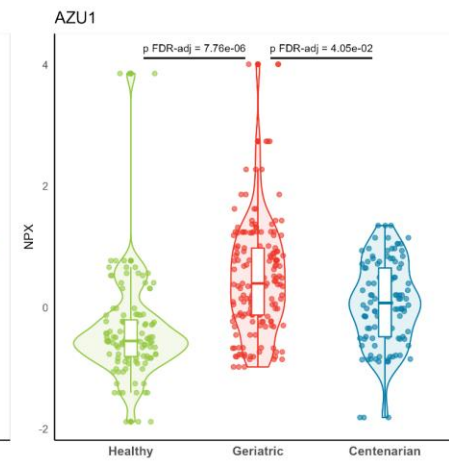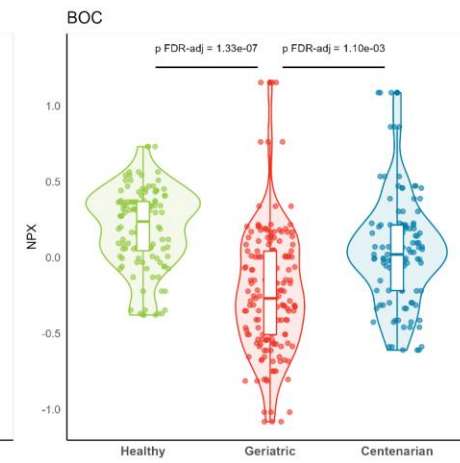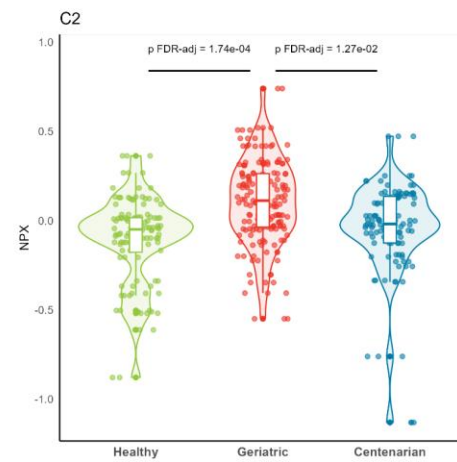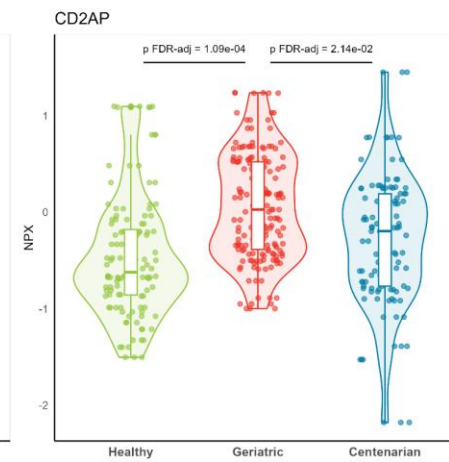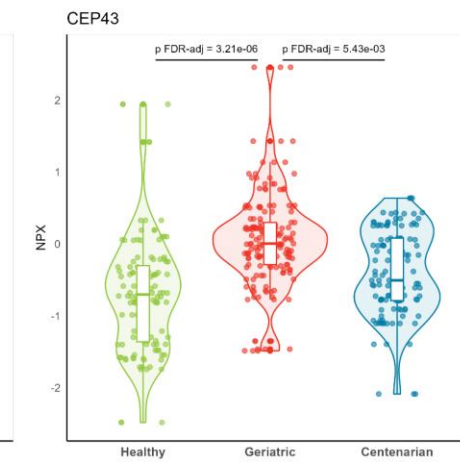

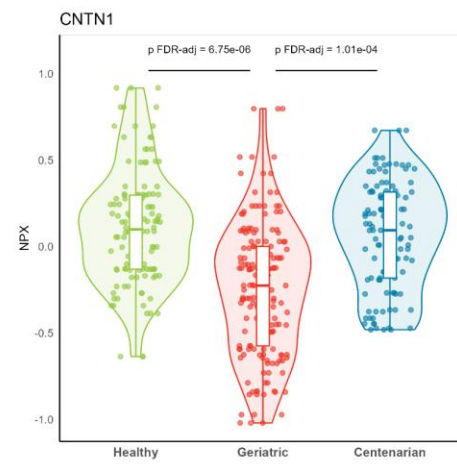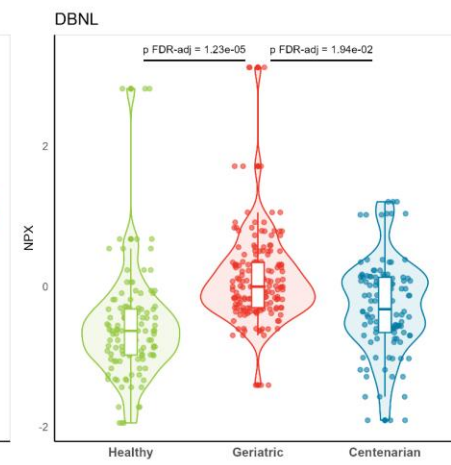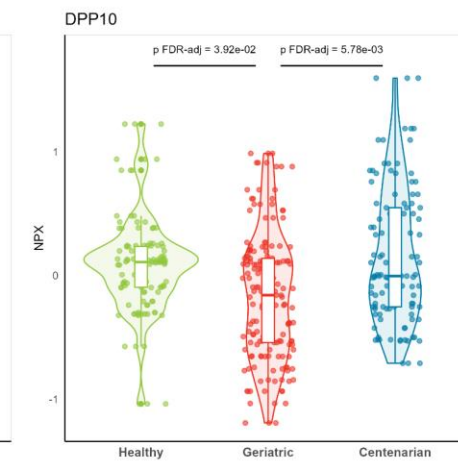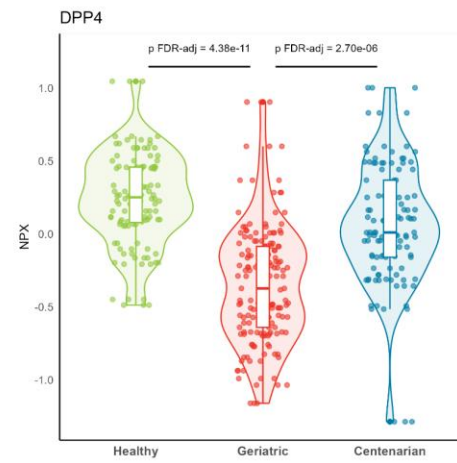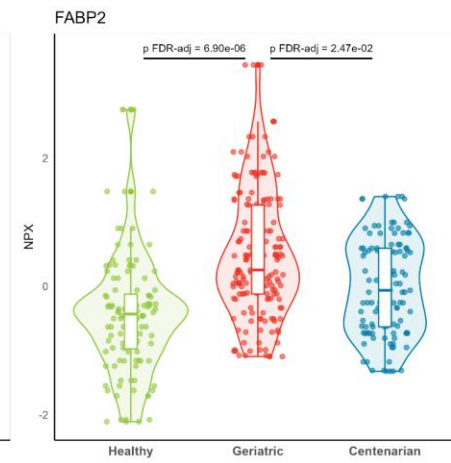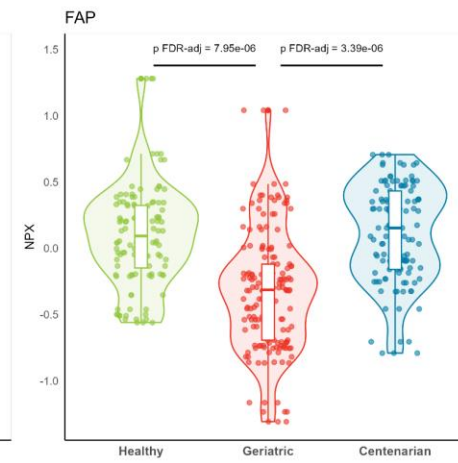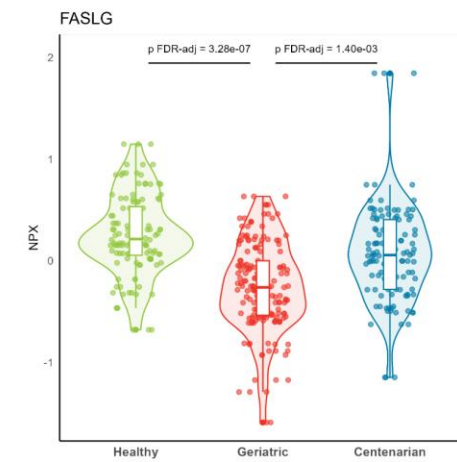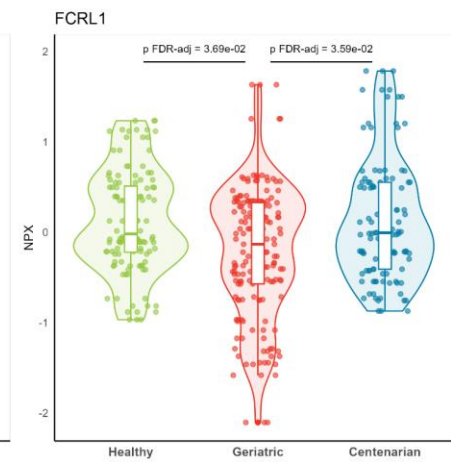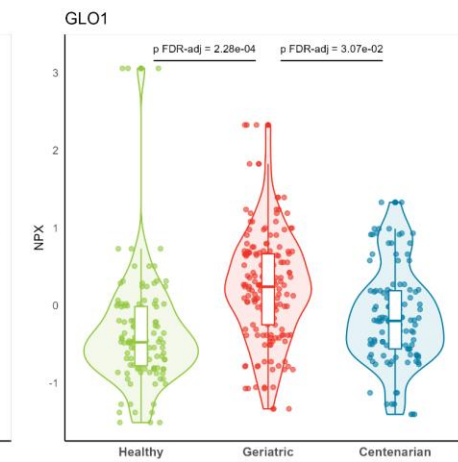

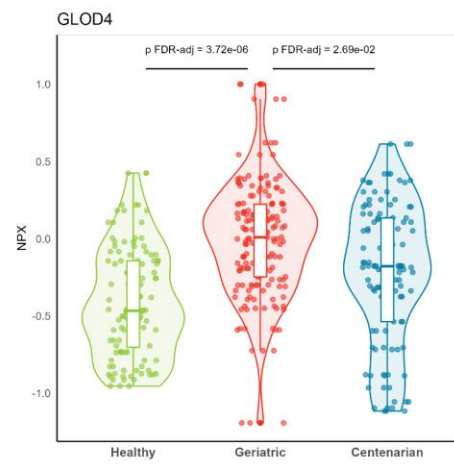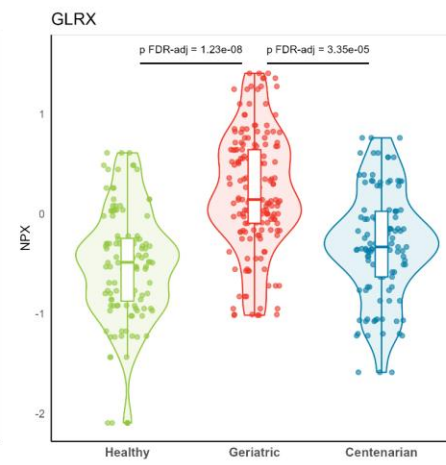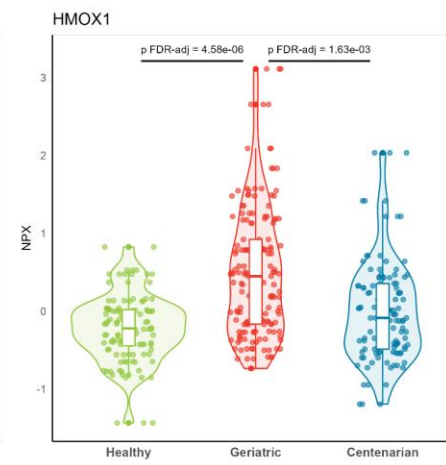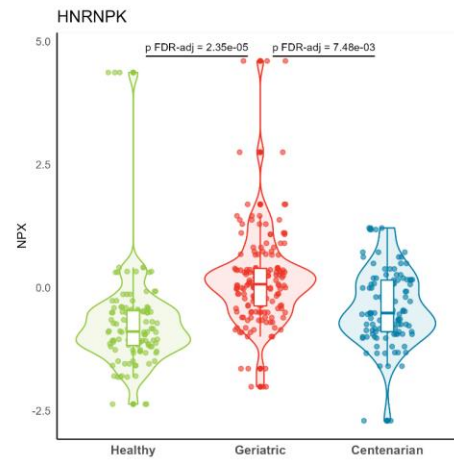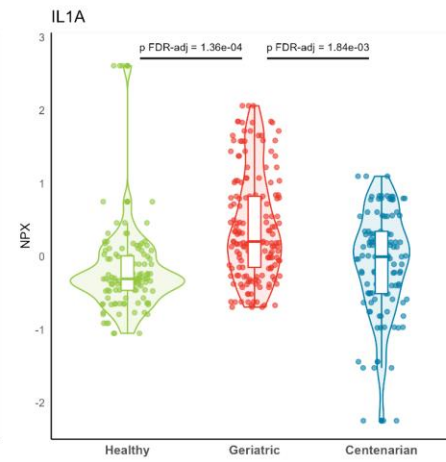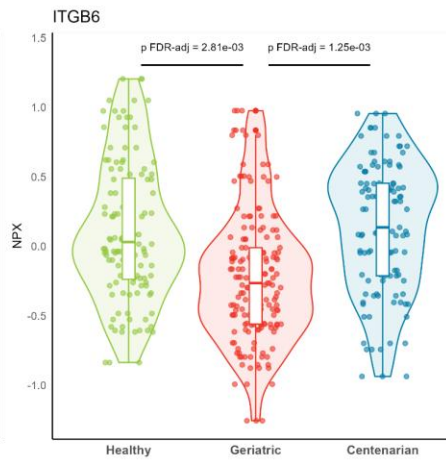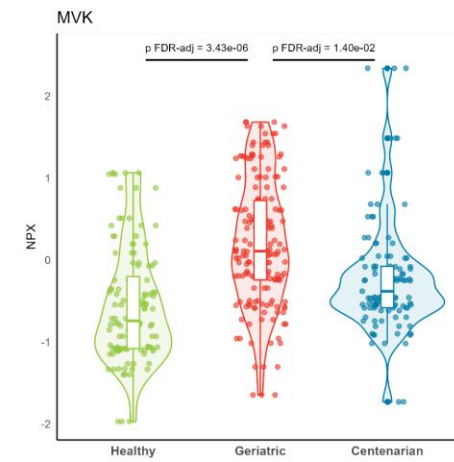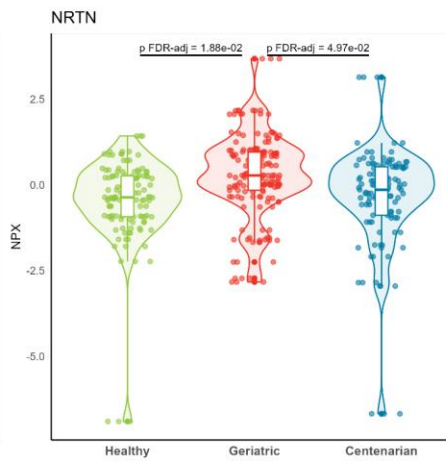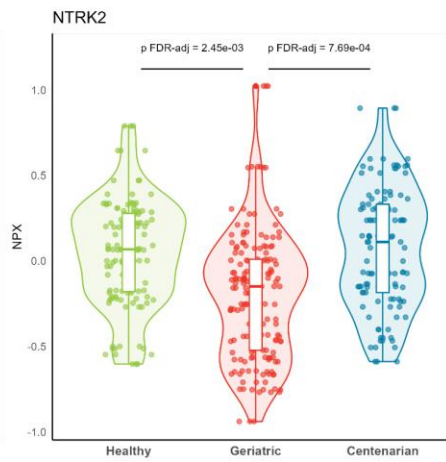

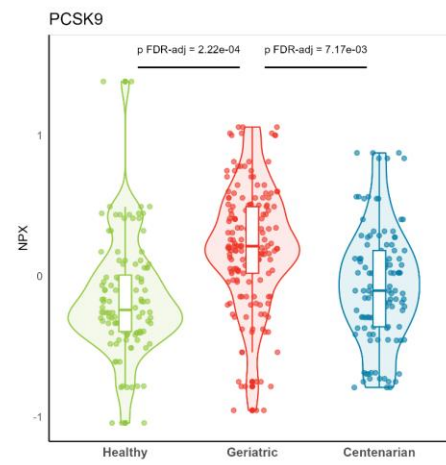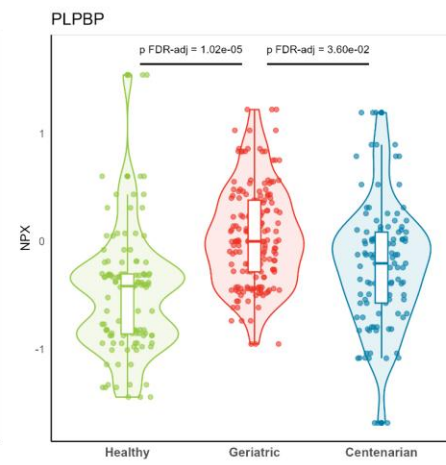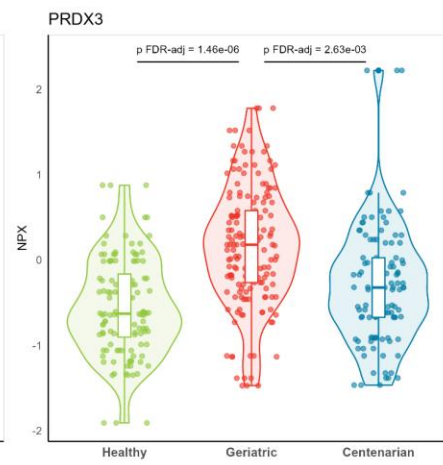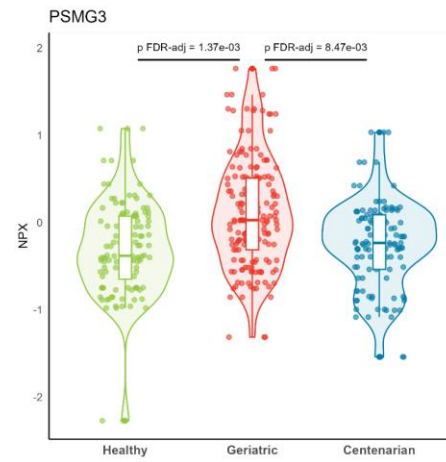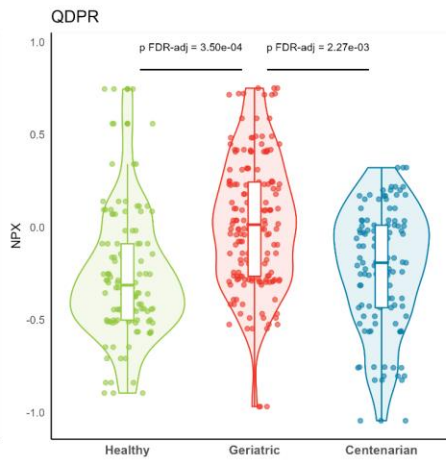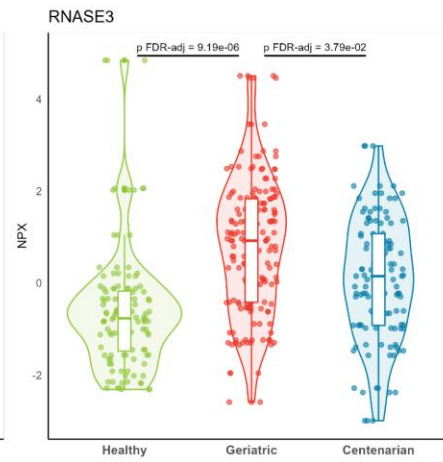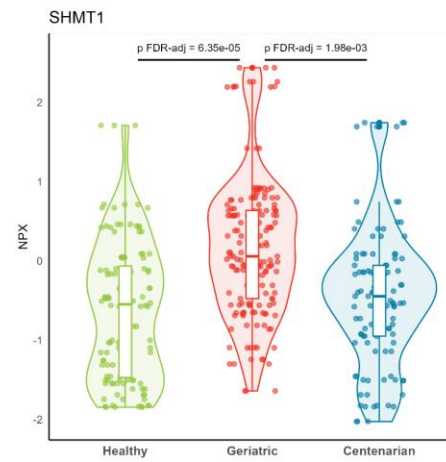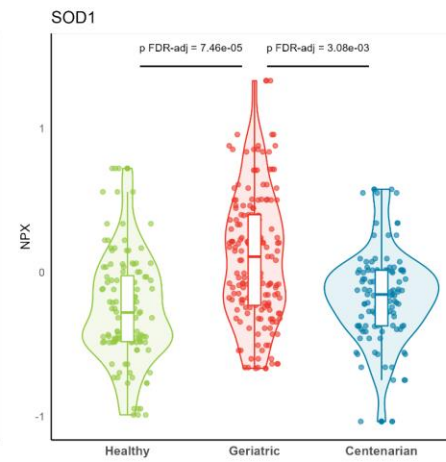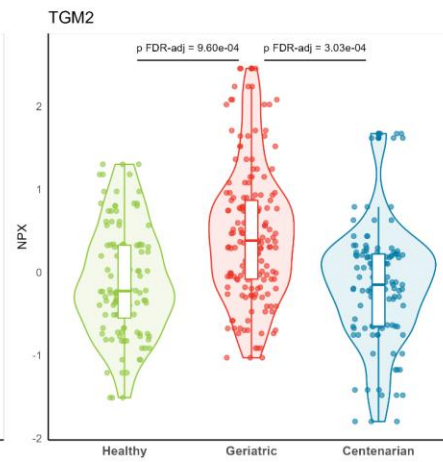

TPP1

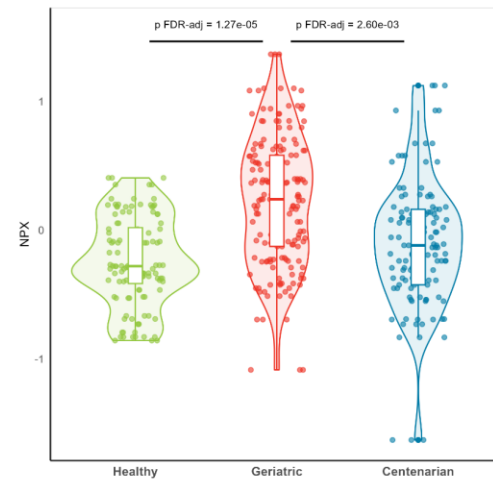

Supplement: Supplementary file 3 — Figure S3: Plasma protein levels for the 37 proteins included in the centenarian youth proteins list. The analysis was performed on ANOVA; proteins significantly different between centenarians and geriatric but similar to healthy individuals were selected. Violin plot showing the relative concentrations between the three groups (healthy, geriatric and centenarian). Bottom and top lines of the boxes depict 25th and 75th percentiles; each dot indicates an individual participant. Log2 fold change and significance levels between groups were calculated using the ANOVA F‐test. If p < 0.05, paired comparisons were conducted with the estimated marginal means. Multiple testing correction was performed using the Benjamini–Hochberg method and a 5% FDR used for the significance threshold. For the final list of centenarian youth proteins with log2 FC and adjusted p‐value for comparisons with Cent2Geriatric see Table S2g. [file ACEL-25-e70409-s002.pdf]
